# In addition to cryptochrome 2, magnetic particles with olfactory co-receptor are important for magnetic orientation in termites

**DOI:** 10.1038/s42003-021-02661-6

**Published:** 2021-09-23

**Authors:** Yongyong Gao, Ping Wen, Ring T. Cardé, Huan Xu, Qiuying Huang

**Affiliations:** 1 Hubei Insect Resources Utilization and Sustainable Pest Management Key Laboratory, College of Plant Science and Technology, Huazhong Agricultural University, Wuhan, 430070 Hubei China; 2 CAS Key Laboratory of Tropical Forest Ecology, Xishuangbanna Tropical Botanical Garden, Chinese Academy of Sciences, Kunming, Yunnan Province 650223 China; 3 Department of Entomology, University of California Riverside, Riverside, CA 92521 USA

**Keywords:** Entomology, Animal behaviour, RNAi

## Abstract

The volatile trail pheromone is an ephemeral chemical cue, whereas the geomagnetic field (GMF) provides a stable positional reference. However, it is unclear whether and how the cryptic termites perceive the GMF for orientation in light or darkness until now. Here, we found that the two termite species, *Reticulitermes chinensis* and *Odontotermes formosanus*, use the GMF for orientation. Our silencing *cryptochrome* 2 (*Cry*2) impaired magnetic orientation in white light but had no significant impact in complete darkness, suggesting that *Cry*2 can mediate magnetic orientation in termites only under light. Coincidentally, the presence of magnetic particles enabled the magnetic orientation of termites in darkness. When knock-downing the olfactory co-receptor (*Orco*) to exclude the effect of trail pheromone, unexpectedly, we found that the *Orco* participated in termite magnetic orientation under both light and darkness. Our findings revealed a novel magnetoreception model depending on the joint action of radical pair, magnetic particle, and olfactory co-receptor.

## Abstract (web summary)

Gao et al. analyze the role of magnetoreceptor candidates cryptochrome 2 (Cry2), magnetic particles and olfactory coreceptor (Orco) in magnetic orientation in two termite species. They report that termites use Cry2 for directional preference in white light, magnetic particles in darkness, and Orco participates in termite magnetic orientation under both light and darkness.

## Introduction

Many organisms (mammals, birds, fishes, and insects) use the geomagnetic field (GMF) to orient and navigate^1–3^. For example, mole rats (*Spalax ehrenbergi*)^4^, spiny lobsters (*Panulirus argus*)^5^, and European robins (*Erithacus rubecula*)^6^ use geomagnetic information to return home and navigate. Monarch butterflies (*Danaus plexippus*)^7,8^ and Bogong moths (*Agrotis infusa*)^9^ use the GMF to steer their migratory flight behavior. Desert ants (*Cataglyphis*)^10^ and leafcutter ants (*Atta colombica*)^11^ use the GMF to orient a vector nest. In the termite *Neocapritermes opacus*, the GMF affects nest construction and foraging for food^12^. Some insects preferentially used the horizontal component of the GMF for orientation^13^. For example, ants use the horizontal component of the GMF to determine the direction of the nest^10^. Therefore, orientation and navigation by the GMF play important roles in animal homing and foraging.

Trail pheromones guide termites from the nest to the food source and return and regulate recruitment^14^. (*Z*, *Z*, *E*)-dodeca-3,6,8-trien-1-ol (DTE) is a trail pheromone of *Reticulitermes* that evaporates quickly without reinforcement in an open arena^15^. Some termite pheromone trails that contain more persistent compounds or are confined to a tunnel can remain active for hours to days without reinforcement^16^, as observed for the persistent neocembrene produced by *Hospitalitermes hospitalis* (Haviland)^17^, DTE trail tunnels in *Reticulitermes flavipes* (Kollar)^18^ and (*Z*)-dodeca-3-en-1-ol trail tunnels in *Macrotermes michaelseni* (Sjöstedt)^19^. In addition to radial foraging tunnels, termites exhibit complex trail networks within their highly branched nest structures. Volatile trail pheromones are not a reliable cue for positioning, so termites may use other cues for orientation. Thirty years ago, a simple test showed that the homeward direction was affected by the artificial magnetic field (AMF) in the termite *Trinermtermes geminatus* (Wasman)^20^. Because of technical restrictions, this previous study only used the distribution of termite numbers to describe magnetic orientation in termites. However, it is unclear whether and how the cryptic termites perceive the GMF for orientation in light or darkness until now.

Some organisms have been shown to be able to perceive the GMF^2,21^, but the sensory mechanism involved is poorly understood. The two main hypotheses are magnetic-particle-based magnetoreception (magnetite hypothesis) and radical-pair-based magnetoreception (radical pair hypothesis)^22–25^. Magnetite hypothesis is that the magnetic field is detected by nanocrystals of magnetite (Fe_3_O_4_), with magnetic remanence as part of a sensory system that signals the movement of these minerals in relation to the magnetic field^25,26^. This movement could potentially be used to detect the intensity, inclination or polarity of the magnetic field. The previous studies showed that mole rats, fish, and sea turtles used the GMF for orientation in complete darkness, which suggested that magnetic orientation behavior might be triggered by magnetic-particle-based magnetoreception mechanisms^4,21,27^. However, the other studies demonstrated that the magnetic field was perceived by light-sensitive chemical reactions involved in the blue-light photoreceptor protein cryptochrome (Cry), which is the only candidate radical pair magnetoreceptor^28^. Cry proteins are known for their roles in the regulation of magnetosensitivity^8,29,30^. For example, Cry-deficient fruit flies are unable to respond to an AMF under full-spectrum light, but the ability to respond to an AMF can be rescued by transferring human *Cry*2 into Cry-deficient fruit flies^31^. The *Cry*2 silencing impairs the ability to detect direction under an AMF in cockroaches under 365 nm UV light^32^. These examples indicate that *Cry* plays a key role in magnetic sensitivity under light. The previous study showed that *Cry*1 (Drosophila-like type 1 *Cry*) was lacking in multiple species of termites and cockroaches, and only *Cry*2 (vertebrate-like type 2 *Cry*) were involved in light-dependent magnetoreception in cockroaches phylogenetically close to termites^32^. However, it remains unknown whether *Cry*2 can mediate magnetic orientation in cryptic termites under light or darkness.

Recently, A magnetic receptor (MagR) has been identified in fruit flies, which can combine with Cry to form a MagR/Cry magnetosensor complex, namely as a biological compass to detect the changes of the magnetic field direction^33^. It provides a new clue to explore the mechanism for biological sensing of magnetic field. However, a theoretical physics calculation suggested that the number of iron atoms in the MagR/Cry complex may be not enough for sensing the magnetic field^34^. In addition, until now, the role of MagR in insects for magnetoreception has not been behaviorally testified.

Termites are similar to many ant species^35,36^, and their olfactory system is crucial for orientation and navigation^37,38^. The olfactory co-receptor (*Orco*) is essential for insect olfaction^39^. For example, odorant detection is impaired in adult *Orco*-mutant fruit flies^40^. Silencing *Orco* impaired the ability to perceive trail pheromones and altered locomotion behavior during following pheromone trails in the termites *Reticulitermes chinensis* Snyder and *Odontotermes formosanus* (Shiraki)^41^. Downregulation of *Orco* reduces the nestmate discrimination ability of the termite *O. formosanus*^42^. The *Orco-*silenced rice planthoppers, *Nilaparvata lugens* (Stal) were found to be unable to seek or locate rice plants in behavioral tests, suggesting that *Orco* is essential in olfactory signal processing in rice planthoppers^43^. A previous study showed that olfaction might be involved in monarch southward migration behaviors at night^7^. However, it is unclear whether *Orco* takes part in mediating orientation in termites.

In order to investigate whether and how the cryptic termites perceive the GMF for orientation in light or darkness, we firstly tested the magnetic orientation of non-fungus-growing termite *R. chinensis* and fungus-growing termite *O. formosanus* under different strengths and directions of the horizontal component of the magnetic field using the EthoVision video-tracking system. *O. formosanus* can forage 100 meters away from the nest, while *R. chinensis* can usually forage within 30 meters^44–46^. Secondly, we used RNA interference to analyze the influence of *Cry*2, *MagR* and *Orco* on the magnetic orientation of the two termite species. Thirdly, we tried to detect the presence of magnetic materials such as iron oxide or other magnetite. Finally, we used orthogonal design tests to analyze the comprehensive effects of *Orco*, *Cry*2, trail pheromone, and magnetic field strength on the orientation of termites. Our findings found a novel magnetoreception model depending on the joint action of radical pair, magnetic particle, and olfactory co-receptor.

## Results

### Termites can use the GMF for orientation in white light and complete darkness

**Fig. 1:**
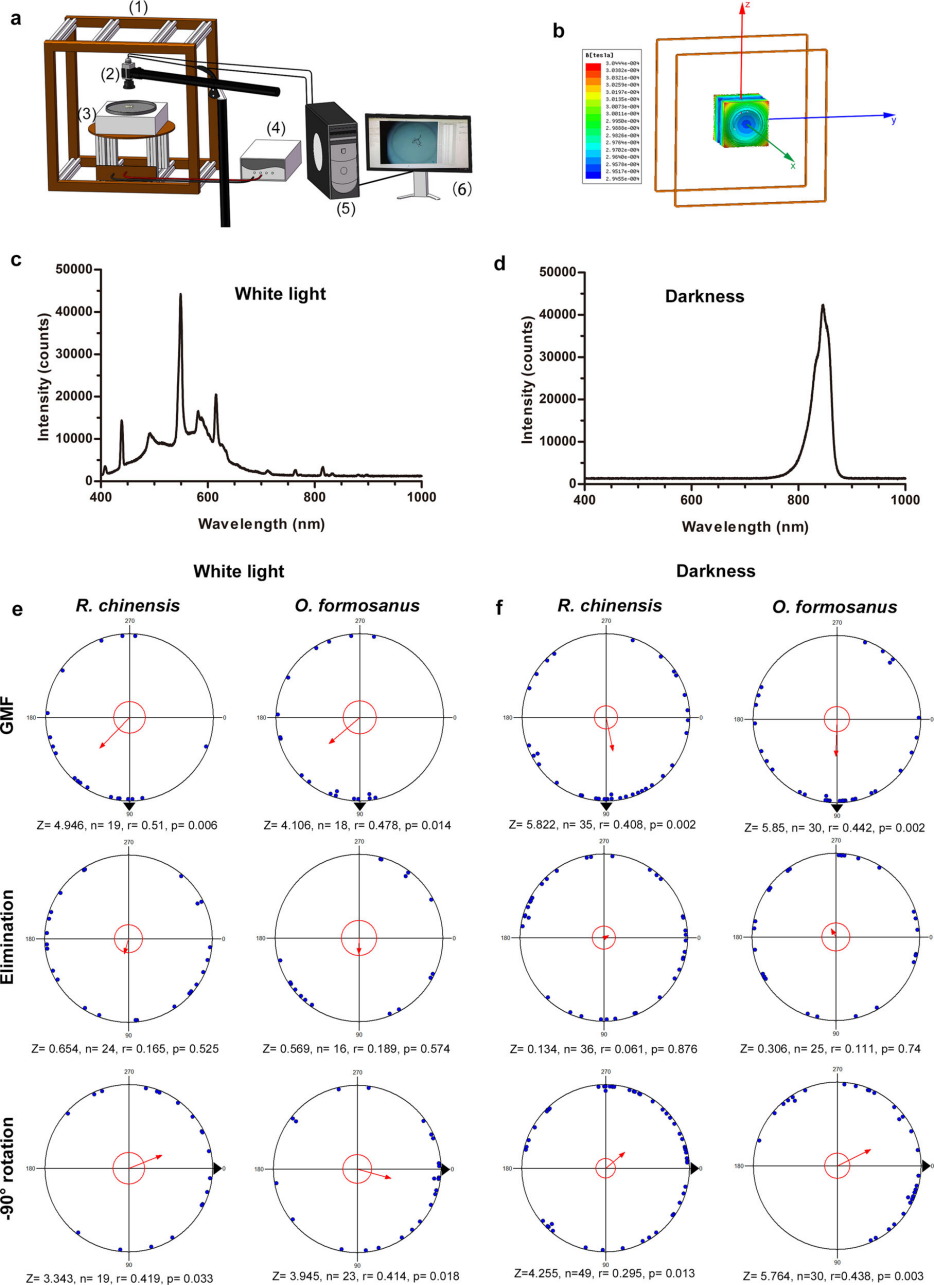
Termites orient with respect to the direction of the horizontal component of the magnetic field in white light and complete darkness. **a** Experimental setup in the laboratory: (1) Helmholtz two-coil system, (2) camera, (3) experimental platform, (4) DC power, (5) EthoVision video-tracking system, and (6) computer screen. **b** Diagrams of the homogeneous magnetic field distributions in the center of the Helmholtz two-coil system. **c**, **d** Intensity and wavelength of light for white light (**c**) or complete darkness (**d**) conditions during experiments. **e** Effect of rotation and eliminating the horizontal component of the magnetic field on heading direction in white light. **f** Effect of rotation and eliminating of the horizontal component of the magnetic field on heading direction in complete darkness. In each circular diagram shown in (**e**, **f**), each blue dot represents an individual’s mean heading direction, and the red arrow represent the group’s mean heading direction during the orientation of worker termites. The length of each arrow represents the mean vector length (*r*-value) of the corresponding mean heading direction, and large dark triangles represents the magnetic north position. When *p* < 0.05, there is significant directionality in orientation at the population level, according to the Rayleigh test of uniformity.

To precisely generate different magnetic conditions, we built a Helmholtz two-coil system (Fig. 1a, b). All the experiments were performed under different magnetic conditions in white light (Fig. 1c) or complete darkness (Fig. 1d). We analyzed the heading directions of walking termites under the different magnetic conditions in white light and complete darkness. The termites showed directional preference under the GMF (Rayleigh test, White light: *R. chinensis*, *r* = 0.51, *p* = 0.006, *O. formosanus*: *r* = 0.478, *p* = 0.014; Darkness: *R. chinensis*, *r* = 0.408, *p* = 0.002, *O. formosanus*: *r* = 0.442, *p* = 0.002) (Fig. 1e, f). However, after the horizontal component of the GMF was eliminated, the directional preference of the termites disappeared (Rayleigh test, White light: *R. chinensis*, *r* = 0.165, *p* = 0.525, *O. formosanus*: *r* = 0.189, *p* = 0.574; Darkness: *R. chinensis*, *r* = 0.061, *p* = 0.876, *O. formosanus*: *r* = 0.111, *p* = 0.74) (Fig. 1e, f). Thus, we concluded that the horizontal component of the GMF provides the necessary cue used by the two termite species to adjust their heading direction during walking in white light and complete darkness. We further found that the directional preference of the termites changed when the horizontal component of the magnetic field was rotated. When the horizontal component of the magnetic field was rotated 90° relative to the GMF orientation, the directional preference of the termites changed (Rayleigh test, White light: *R. chinensis*, *r* = 0.419, *p* = 0.033, *O. formosanus*, *r* = 0.414, *p* = 0.018; Darkness: *R. chinensis*, *r* = 0.295, *p* = 0.013, *O. formosanus*, *r* = 0.438, *p* = 0.003) (Fig. 1e, f).

### Magnetic orientation is not affected by a radial pheromone maze

**Fig. 2:**
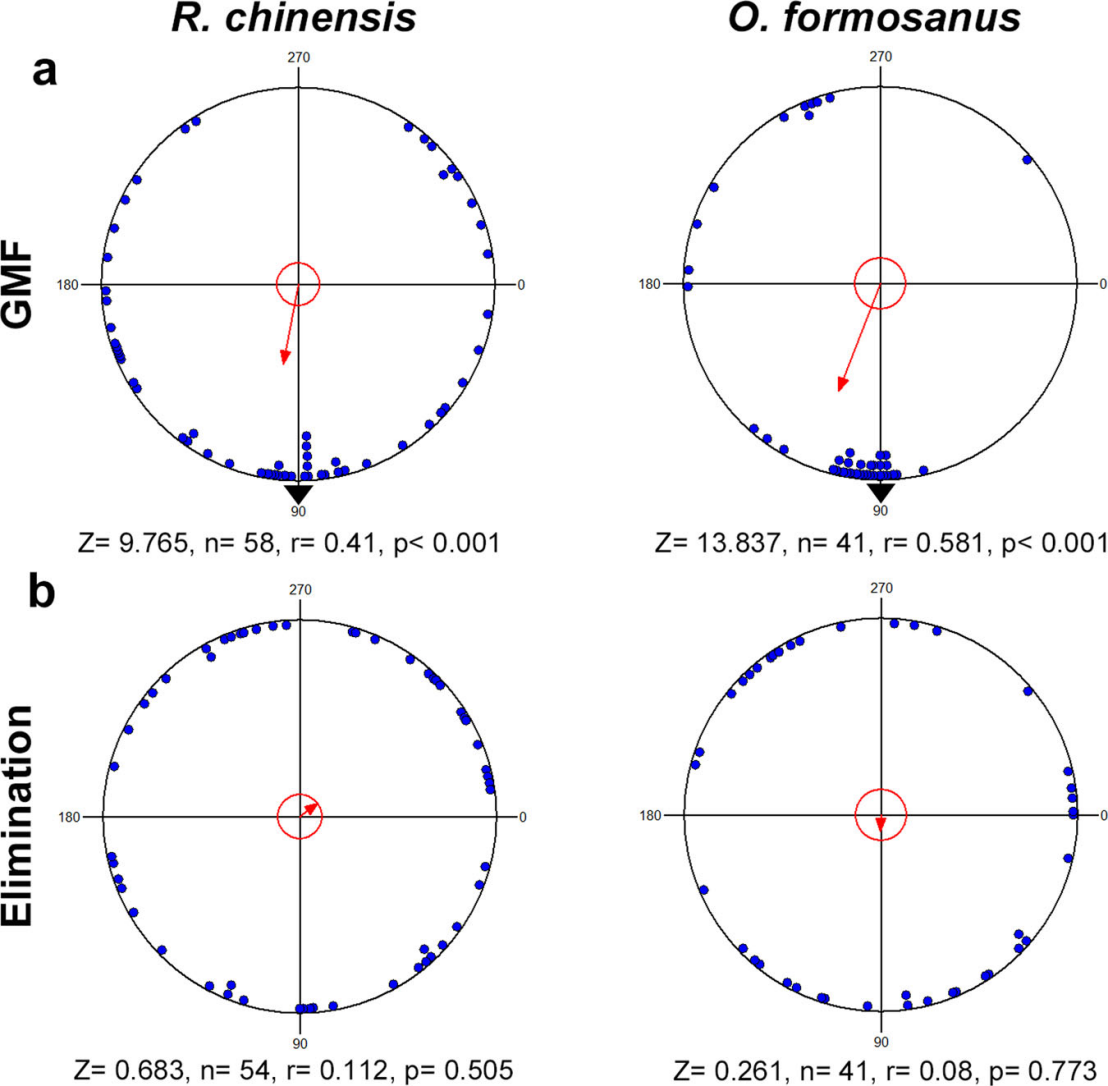
Termites orient with respect to the direction of the GMF in a radial pheromone maze. **a** Effect of the GMF on heading direction in a radial pheromone maze. **b** Effect of eliminating the horizontal component of the magnetic field on heading direction in a radial pheromone maze.

In choice tests, the termites followed DTE or DDE trails, based on the trail-following bioassay results. In *R. chinensis*, the highest trail-following activity was observed in the presence of 10 or 0.1 pg/cm for DTE (Supplementary Table 1). For *O. formosanus*, the highest trail-following activity was observed in the presence of 0.1 or 0.01 pg/cm for DDE (Supplementary Table 2). We tested magnetic orientation in the eight-arm radial pheromone maze in white light. The two termite species exhibited directional preference under the GMF (Rayleigh test, *R. chinensis*: *r* = 0.41, *p* < 0.001; *O. formosanus*: *r* = 0.581, *p* < 0.001) (Fig. 2a). When the horizontal component of the GMF was eliminated, the directional preference of the two termite species was abolished in white light (Rayleigh test, *R. chinensis*: *r* = 0.112, *p* = 0.505 > 0.05; *O. formosanus*: *r* = 0.08, *p* = 0.773 > 0.05) (Fig. 2b).

### The effect of *Cry2* and *MagR* knockdown on magnetic orientation in white light and complete darkness

**Fig. 3:**
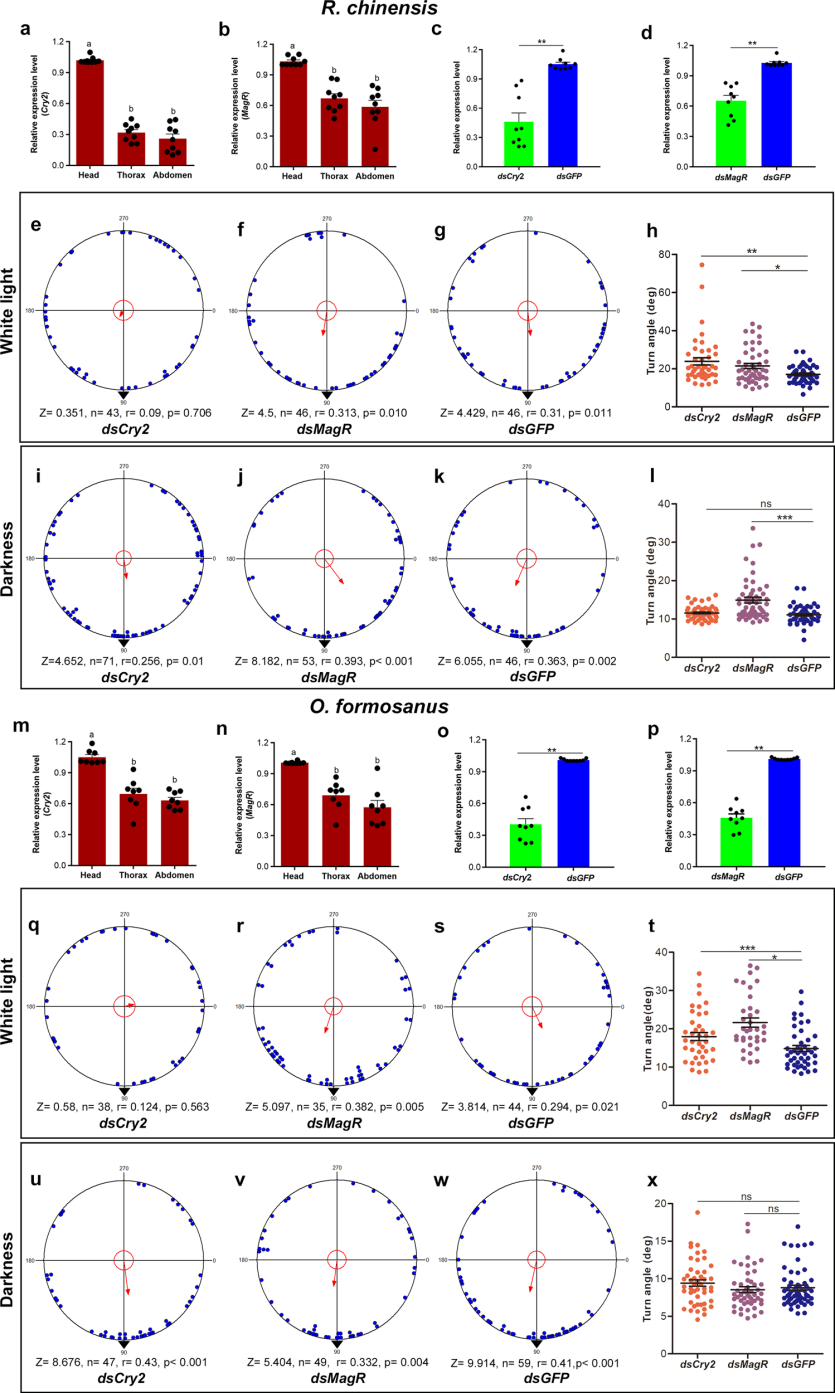
Knockdown of Cry2 abolishes the directional preference of *R. chinensis* and *O. formosanus* under the GMF in white light. **a**, **b** We confirmed the relative expression levels of the *Cry*2 (**a**) and *MagR* (**b**) genes in different organs of *R. chinensis*. **c**, **d** *Cry*2 (**c**) and *MagR* (**d**) mRNA levels in *R. chinensis* heads 3 days after injecting ds*RNAs*. **e**–**g** Orientation behavior of *R. chinensis* in the GMF 3 days after the injection of ds*Cry*2 (**e**), ds*MagR* (**f**), or ds*GFP* (**g**) in white light. **h** The walking turn angle of the *R. chinensis* injected with ds*Cry*2, ds*MagR*, or ds*GFP* was assessed 3 days after injection in white light. **i**–**k** Orientation behavior of *R. chinensis* in the GMF 3 days after the injection of ds*Cry*2 (**i**), ds*MagR* (**j**) or ds*GFP* (**k**) in complete darkness. **l** The walking turn angle of the *R. chinensis* injected with ds*Cry*2, ds*MagR* or ds*GFP* was assessed 3 days after injection in complete darkness. **m**–**n** We confirmed the relative expression levels of the *Cry*2 (**m**) and *MagR* (**n**) genes in different tissues of *O. formosanus*. Each value represents the mean ± SEM, and lowercase letters above the bars indicate significant differences between body parts according to Tukey’s HSD tests (**a**, **b**, **m**, **n**). **o**, **p** *Cry*2 (**o**) and *MagR* (**p**) mRNA levels in *O. formosanus* heads 3 days after the injection of ds*RNAs*. Bars represent the mean ± SEM. Asterisks indicate significant differences determined by the Wilcoxon test (**p < 0.01) (**c**, **d**, **o**, **p**). **q**–**s** Orientation behavior of *O. formosanus* in the GMF 3 days after the injection of ds*Cry*2 (**q**), ds*MagR* (**r**), or ds*GFP* (**s**) in white light. **t** The walking turn angle of the *O. formosanus* injected with ds*Cry*2, ds*MagR* or ds*GFP* was assessed 3 days after injection in white light. **u**–**w** Orientation behavior of *O. formosanus* in the GMF 3 days after the injection of ds*Cry*2 (**u**), ds*MagR* (**v**), or ds*GFP* (**w**) in complete darkness. **x** The walking turn angle of the *O. formosanus* injected with ds*Cry*2, ds*MagR*, or ds*GFP* was assessed 3 days after injection in complete darkness. Data are represented as the mean ± SEM. Asterisks indicate significant differences determined (*p < 0.05; **p < 0.01; ***p < 0.001), and “ns” indicates was not significant by the Mann–Whitney test (**h**, **l**, **t**, **x**).

The qRT-PCR results for *Cry*2 and *MagR* in the head, thorax and abdomen of the two termite species demonstrated that *Cry*2 and *MagR* were expressed in all the examined body parts (Fig. 3a, b, m and n). The expression of *Cry*2 and *MagR* in the two termite species significantly differed (*ANOVA*, *R. chinensis*: *Cry*2, *F*_(2,24)_ *=* 59.84; *p* < 0.001; *MagR*, *F*_(2,24)_ *=* 20.16, *p* < 0.001; *O. formosanus*: *Cry*2, *F*_(2,21)_ *=* 34.19, *p* < 0.001; *MagR*, *F*_(2,21)_ *=* 21.20, *p* < 0.001) between different organs, and the head of the termites exhibited the highest levels of transcription (Fig. 3a, b, m and n). The injection of (ds) *Cry*2 or *MagR* caused a significant decrease in the *Cry*2 or *MagR* mRNA level in the heads of the two termite species (Wilcoxon test, *R. chinensis*: *Cry*2, *Z* = −2.67*, n* = 9, *p* < 0.01, *MagR, Z* = −2.67, *n* = 9, *p* < 0.01; *O. formosanus*: *Cry*2, *Z* = −2.67, *n* = 9, *p* < 0.01, *MagR*, *Z* = −2.67, *n* = 9, *p* < 0.01) (Fig. 3c, d, o and p), suggesting that the *Cry*2 and *MagR* genes were significantly knocked down 3 days after injection.

The injection of ds*Cry*2 abolished the directional preference of *R. chinensis* (Rayleigh test, *r* = 0.09, *p* = 0.706) (Fig. 3e, Supplementary Movie 1) and *O. formosanus* (Rayleigh test, *r* = 0.124, *p* = 0.563) (Fig. 3q, Supplementary Movie 2) in white light, but the termites injected with ds*MagR* or ds*GFP* still exhibited directional preference (Rayleigh test, *R. chinensis*: ds*MagR*, *r* = 0.313, *p* = 0.01, *dsGFP*, *r* = 0.31, *p* = 0.011; *O. formosanus*: ds*MagR*, *r* = 0.382, *p* = 0.005, ds*GFP*, *r* = 0.294, *p* = 0.021) (Fig. 3f, g, r and s). The knockdown of *Cry*2 in the termites resulted in a significant increase in the turn angle as compared to control termites (Mann–Whitney test, *R. chinensis*: *Z* = −3.15*, df* = 88*, p* < 0.05; *O. formosanus*: *Z* = −2.43, *df* = 81, *p* < 0.05) (Fig. 3h, t). In addition, the injection of ds*MagR* significantly increased the walking turn angle compared with the control (Mann–Whitney test, *R. chinensis*: *Z* = −2.00, *df* = 90, *p* < 0.05; *O. formosanus*: *Z* = −4.41, *df* = 77, *p* < 0.001) (Fig. 3h, t).

However, the termites injected with ds*Cry*2 exhibited directional preference in complete darkness (Rayleigh test, *R. chinensis*: *r* = 0.256, *p* = 0.01; *O. formosanus*: *r* = 0.43, *p* < 0.001) (Fig. 3i and u), and the termites injected with ds*MagR* or ds*GFP* also exhibited directional preference (Rayleigh test, *R. chinensis*: ds*MagR*, *r* = 0.393, *p* < 0.001, *dsGFP*, *r* = 0.363, *p* = 0.002; *O. formosanus*: ds*MagR*, *r* = 0.332, *p* = 0.004, ds*GFP*, *r* = 0.41, *p* < 0.001) (Fig. 3j, k, v and w). In addition, the injection of ds*MagR* significantly increased the walking turn angle compared with the control in *R. chinensis* (Mann–Whitney test, *Z* = −4.371, *df* = 97, *p* < 0.001) (Fig. 3l), but the injection of ds*MagR* had no effect on the walking turn angle as compared to the control in *O. formosanus* (Mann–Whitney test, *Z* = −0.651, *df* = 106, *p* = 0.515) (Fig. 3x).

### The magnetic particles are present in the two termite species

**Fig. 4:**
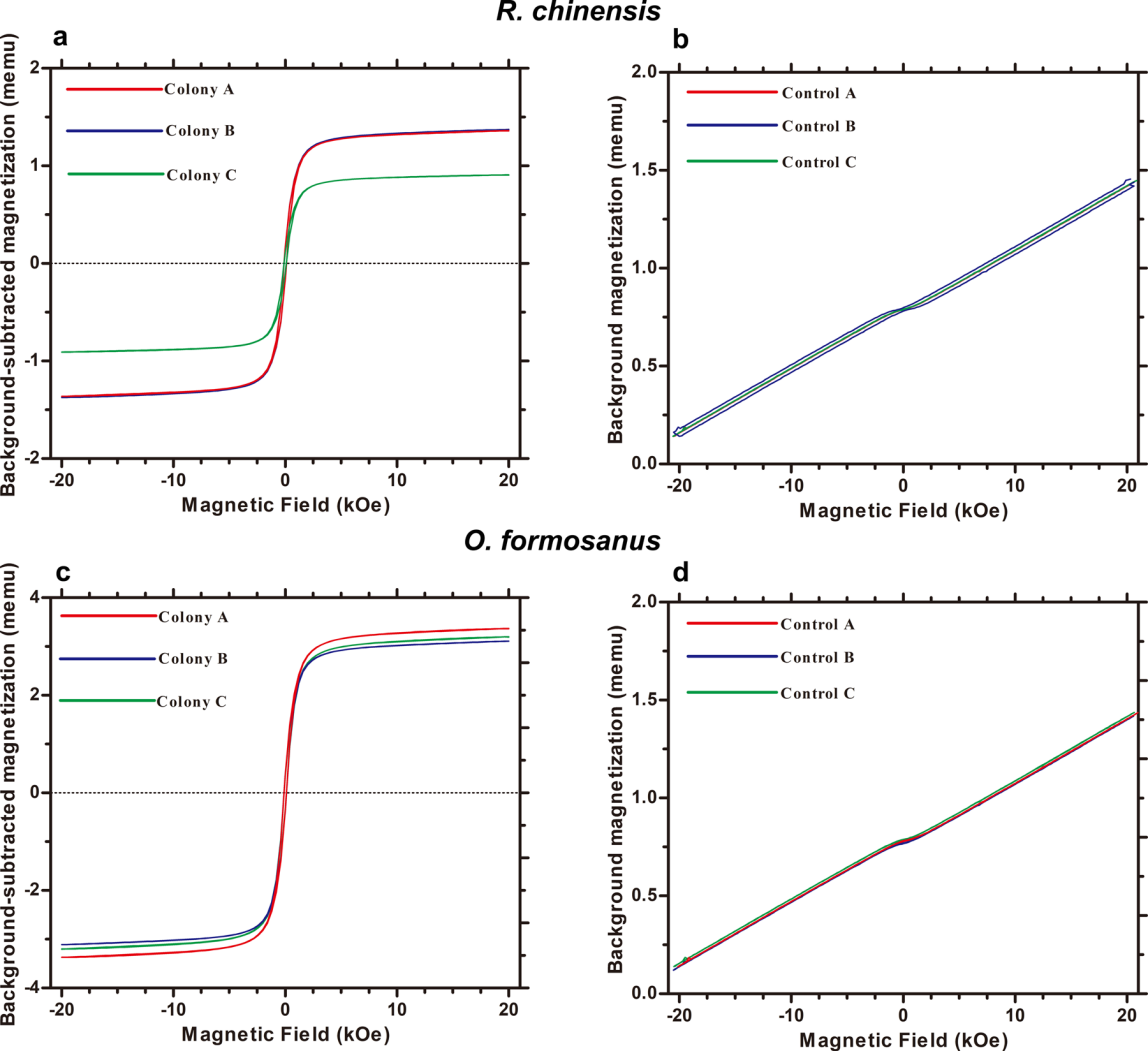
The measurement of magnetic particles using VSM for the workers of two termite species. **a**, **b** Hysteresis curves of worker *R. chinensis* (**a**) and empty cellulose capsules (**b**). **c**, **d** Hysteresis curves of worker *O. formosanus* (**c**) and empty cellulose capsules (**d**).

The magnetic hysteresis curves were obtained by testing *R. chinensis* (Fig. 4a, b) and *O. formosanus* (Fig. 4c, d) tissue with a vibrating sample magnetometer (VSM). The magnetic parameters including saturation magnetization (*Js*), remanent magnetization (*Jrs*), coercive field (*Hc*) and *Jrs*/*Js* ratio for *R. chinensis* and *O. formosanus* were shown in Supplementary Table 3. The S-shaped magnetic hysteresis curves demonstrated the existence of magnetic particles in the tissue of two termite species.

### The knockdown of *Orco* is accompanied by disorientation under the GMF in termites

**Fig. 5:**
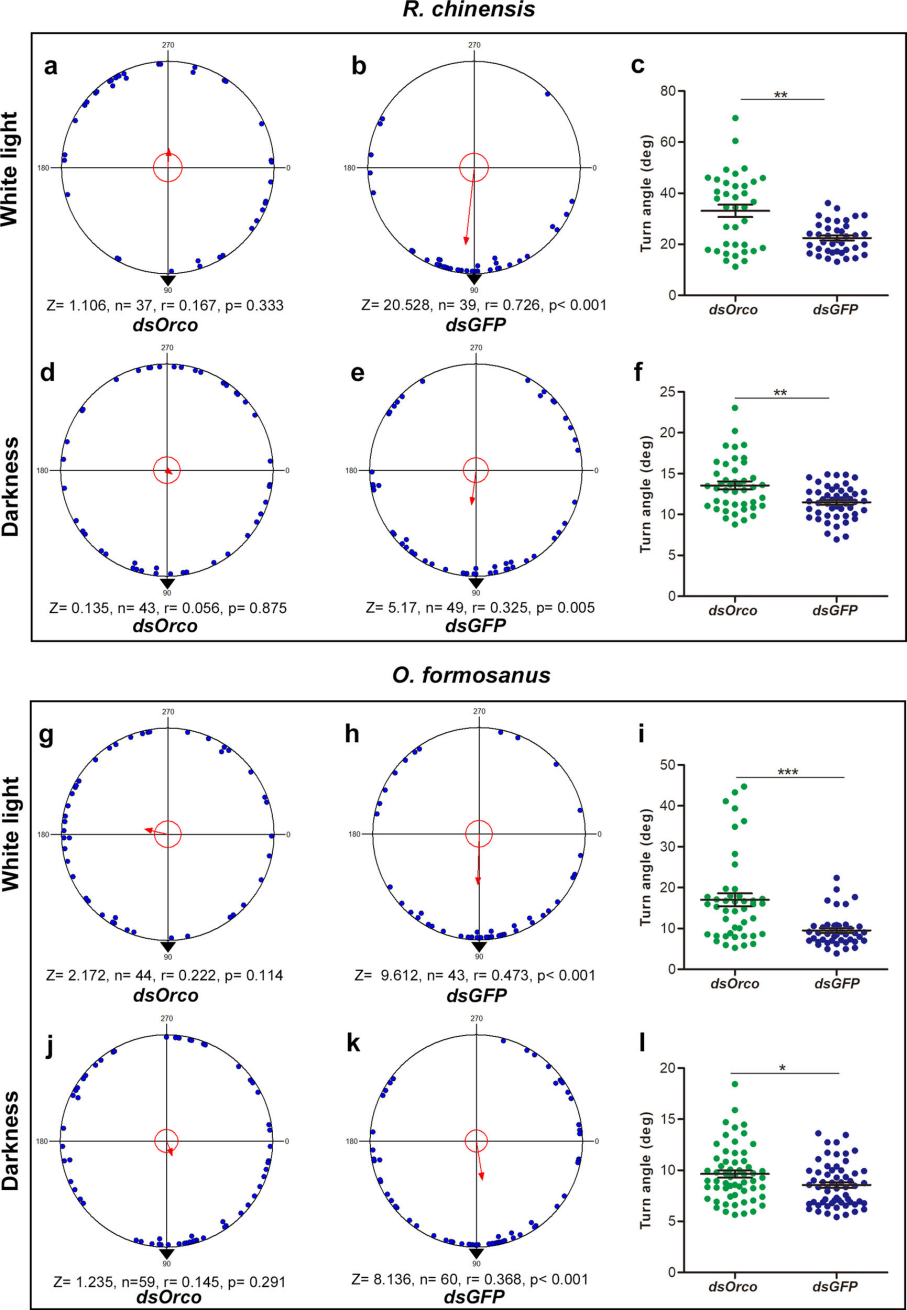
Knockdown of *Orco* abolishes the directional preference of *R. chinensis* and *O. formosanus* under the GMF in white light and complete darkness. **a**, **b** The orientation behavior of *R. chinensis* 5 days after the injection of ds*Orco* (**a**) or ds*GFP* (**b**) in white light. **c** The walking turn angle of the *R. chinensis* injected with ds*Orco* and ds*GFP* was assessed 5 days after injection in white light. **d**, **e** The orientation behavior of *R. chinensis* 5 days after the injection of ds*Orco* (**d**) or ds*GFP* (**e**) in complete darkness. **f** The walking turn angle of the *R. chinensis* injected with ds*Orco* and ds*GFP* was assessed 5 days after injection in complete darkness. **g**, **h** The orientation behavior of *O. formosanus* 3 days after the injection of ds*Orco* (**g**) or ds*GFP* (**h**) in white light. **i** The walking turn angle of the *O. formosanus* injected with ds*Orco* and ds*GFP* was assessed 3 days after injection in white light. **j**, **k** The orientation behavior of *O. formosanus* 3 days after the injection of ds*Orco* (**j**) or ds*GFP* (**k**) in complete darkness. **l** The walking turn angle of the *O. formosanus* injected with ds*Orco* and ds*GFP* was assessed 3 days after injection in complete darkness. The data represent the mean ± SEM. Asterisks indicate significant differences determined by the Mann–Whitney test (*p < 0.05; **p < 0.01; ***p < 0.001) (**c**, **f**, **i**, **l**).

The injection of ds*Orco* caused a significant decrease in *Orco* mRNA levels in the heads of *R. chinensis and O. formosanus* (Wilcoxon test, *R. chinensis*: *Z* = −2.80, *n* = 10, *p* < 0.01; *O. formosanus*: *Z* = −2.52, *n* = 8, *p* < 0.05) (Supplementary Fig. 1a, c), suggesting successful knockdown of the *Orco* gene after injection. We tested whether the knockdown of *Orco* could alter the chemosensory abilities of the two termite species. We found that the injection of ds*Orco* significantly decreased the trail pheromones perception activity in the two termite species (Mann–Whitney test, *R. chinensis*: *Z* = −6.19, *df* = 85, *p* < 0.001; *O. formosanus*: *Z* = −3.15, *df* = 70, *p* < 0.01) (Supplementary Fig. 1b, d). To test the involvement of *Orco* in directional magnetoreception in the termites *R. chinensis* and *O. formosanus*, we carried out behavioral assays under the GMF in white light and complete darkness. The injection of ds*Orco* abolished the directional preference of *R. chinensis* (Rayleigh test, White light: *r* = 0.167, *p* = 0.333; Darkness: *r* = 0.056, *p* = 0.875) (Figs. 5a, d, Supplementary Movie 3) and *O. formosanus* (Rayleigh test, White light: *r* = 0.145, *p* = 0.291; Darkness: *r* = 0.145, *p* = 0.291) (Figs. 5g, j, Supplementary Movie 4) in white light and complete darkness. However, the ds*GFP*-injected termites still exhibited directional preference in white light and complete darkness (Rayleigh test, White light: *R. chinensis*: *r* = 0.726, *p* < 0.001; *O. formosanus*: *r* = 0.473, *p* < 0.001; Darkness: *R. chinensis*: *r* = 0.325, *p* = 0.005, *O. formosanus*: *r* = 0.368, *p* < 0.001) (Fig. 5b, e, h and k). The knockdown of *Orco* resulted in a significant increase in the turn angle compared with the control in *R. chinensis* (Mann–Whitney test, White light: *Z* = −3.08, *df* = 75, *p* < 0.01; Darkness: *Z* = −3.095, *df* = 90, *p* < 0.01) (Fig. 5c, f) and *O. formosanus* (Mann–Whitney test, White light: *Z* = −3.88, *df* = 85, *p* < 0.001; Darkness: *Z* = −2.46, *df* = 117, *p* < 0.05) (Figs. 5i, l) in white light and complete darkness.

### The magnetic field strength, *Orco* and *Cry*2, comprehensively affected the orientation of the termites

**Fig. 6:**
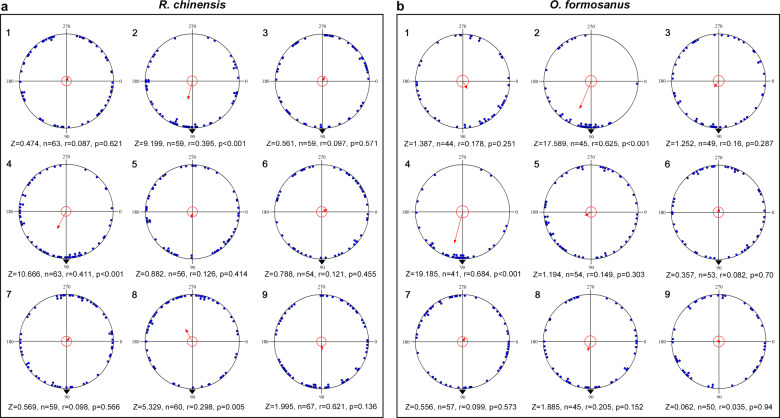
The comprehensive effects of *Orco*, *Cry*2, the pheromone trail and magnetic field strength on the orientation of *R. chinensis* and *O. formosanus*. **a** Heading directions of *R. chinensis* in relation to four factors in the orthogonal design. **b** Heading direction of *O. formosanus* in relation to four factors in the orthogonal design. Numbers on the upper left corner of each schematic indicate different treatments of four factors in the orthogonal design L9 (3^4^) (For information about treatment 1-9 of *R. chinensis* and *O. formosanus*, see Supplementary Table 8 in Materials and Methods).

To explore the comprehensive influence of *Orco*, *Cry*2, the pheromone trail and magnetic field strength on the orientation of the termites, we analyzed the orientation of the two termite species under orthogonal design L_9_ (3^4^). We found that *R. chinensis* showed directional preference under treatments 2 (noninjected, ds*GFP*, 0.01 pg/cm, GMF) (Rayleigh test, *r* = 0.395, *p* < 0.001), 4 (ds*GFP*, noninjected, 0.01 pg/cm, 0.1 mT) (Rayleigh test, *r* = 0.411, *p* < 0.001) and 8 (ds*Orco*, ds*GFP*, 0 pg/cm, 0.1 mT) (Rayleigh test, *r* = 0.298*, p* = 0.005) (Fig. 6a). Similarly, the termite *O. formosanus* exhibited directional preference under treatments 2 (noninjected, ds*GFP*, 0.01 pg/cm, GMF) (Rayleigh test, *r* = 0.625, *p* < 0.001) and 4 (ds*GFP*, noninjected, 0.01 pg/cm, 0.1 mT) (Rayleigh test, *r* = 0.684, *p* < 0.001) (Fig. 6b). However, after the horizontal component of the GMF was eliminated and the *Cry*2 or *Orco* were knocked down, the two termite species became disoriented (Fig. 6a1, 3, 5, 6, 7, 9; Fig. 6b1, 3, 5, 6, 7, 8, 9). These results further demonstrated that magnetic field strength, the *Cry*2 and *Orco* genes could mediate the orientation of the termites.

## Discussion

The GMF provides directional and positional information to many organisms^1,21,47^. The previous studies have shown that tethered oriental armyworm moths (*Mythimna separata*) exhibited a common orientation under the GMF, but they are disoriented in a weak magnetic field (500 nT)^48^. The GMF-navigating desert ants no longer maintained their nest-centered gaze directions over a long interval when the horizontal component of the GMF is eliminated^10^. In this study, the two termite species phylogenetically distant to ants also displayed a significant directional preference under the GMF in white light and complete darkness, but when the horizontal component of the GMF was eliminated, the termites lost their directional preference. Our results suggested that magnetoreception might be a common feature of two termite species. Therefore, we conclude that termites can perceive the GMF cues similarly to migratory moths and desert ants.

The horizontal component of the GMF provides the necessary cue to social insects to adjust their heading direction during walking^10^. For example, the fire ants can consistently change their orientation when the magnetic field is altered in a circular arena^49^. Similarly, the leafcutter ants can alter their preferred direction by 180° when the horizontal component of the magnetic field is reversed^5^. Our behavioral evidence showed that the termites could correspondingly change their directional preference after the horizontal component of the magnetic field was rotated. It is well known that the trail pheromones of termites play a clear role in orientation^16,38^, but volatile trail pheromones are not a reliable cue for positioning, so termites may use other cues for orientation. Based on the present results, we conclude that the GMF plays an important role in the orientation of termites especially when trail pheromones cannot provide a precise direction.

Cry has been reported as the key component of the light-dependent biological compass relying on the radical pair magnetoreceptor mechanism^50–52^. In transgenic fruit flies, the Cry protein was found to be involved in magnetic-sensitive behavior^50^. RNA interference tests showed that the *Cry*2 could detect direction under an AMF in cockroaches phylogenetically close to termites under 365 nm UV light^32^. These studies indicated that the Cry protein can sense the magnetic field in light^53^. In this study, we detected the substantial expression of *Cry*2, and found that the knockdown of *Cry*2 resulted in severe deficiencies of directional preference and a significant increase in the turn angle under the GMF in white light in the two termite species. Termites foraging under galleries can receive white light through the cracks and pores (Supplementary Fig. 2)^54^. Therefore, the light-dependent *Cry*2 plays the same role in the perception of the magnetic field in termites as the above fruit fly and cockroaches, which support the hypothesis of radical-pair-based magnetoreception^23^.

Termites can use the GMF for orientation in complete darkness. Expectedly, the knockdown of *Cry*2 had no significant effect on directional preference of two termite species under the GMF in complete darkness, indicating that the *Cry*2 cannot play its role in magnetic orientation without light in termites^30^. Similarly, the Cry2-mediated magnetoreception requires the participation of light in cockroaches^32,55^. However, what mechanism does the termite use to perceive the GMF for orientation in complete darkness? The previous studies hypothesized that the several animals (mole rats, fish and sea turtles) might use the magnetic-particles for magnetic orientation in darkness^4,47,56^, but the existence of magnetic particles were not confirmed in these studies. In this study, we detected the presence of magnetic particles in termites, indicating that termites may use magnetic particles for magnetic orientation in darkness, which supports the hypothesis of the magnetic-particle-based magnetoreception^21,27,57^. Given the evidence for the coexistence of two magnetoreception mechanisms that has been reported for several birds^58,59^, these data indicate that both radical-pair-based and magnetic-particle-based magnetoreception mechanisms may co-exist for magnetic orientation in termites.

The *MagR* has been expressed in fruit flies^33^, fish^60^, moths^61^, and termites here. Although *MagR* may act jointly with *Cry*2, its magnetoreception function is unsolved^62^. In the two termite species studied here, the knockdown of *MagR* had no significant effect on directional preference under the GMF, but significantly increased the turn angle during walking in white light, suggesting that *MagR* may affect the behavior of termites in magnetic fields.

The *Orco* gene is involved in the magnetic orientation in both light and darkness. A previous study showed that olfaction may be involved in monarch southward migration behaviors at night^7^. In this study, the knockdown of *Orco* in termites impaired the magnetic orientation in white light and complete darkness, which suggested that *Orco* may took part in perceiving the GMF for orientation in termites. Generally, the *Orco* is involved in olfaction-based orientation, and antennae are the main olfactory organ of insects. The previous studies have strongly suggested that antennae are involved in monarch magnetoreception^7,8,63^. In this study, *Orco* silencing damaged the olfactory system of the termites and thereby affected their magnetic orientation, suggesting that antennae may be needed for termite magnetoreception. According to our knowledge, it is the first time to report that *Orco* participates in the GMF orientation both in light and darkness. Certainly, anyone of the *Cry*2, magnetic particles, and *Orco* is unable to let termites orientate normally in the GMF, independently. Reasonably, these factors involved in magnetic orientation should function jointly with the changing environmental conditions, such as light condition, locomotion sites, etc.

**Fig. 7:**
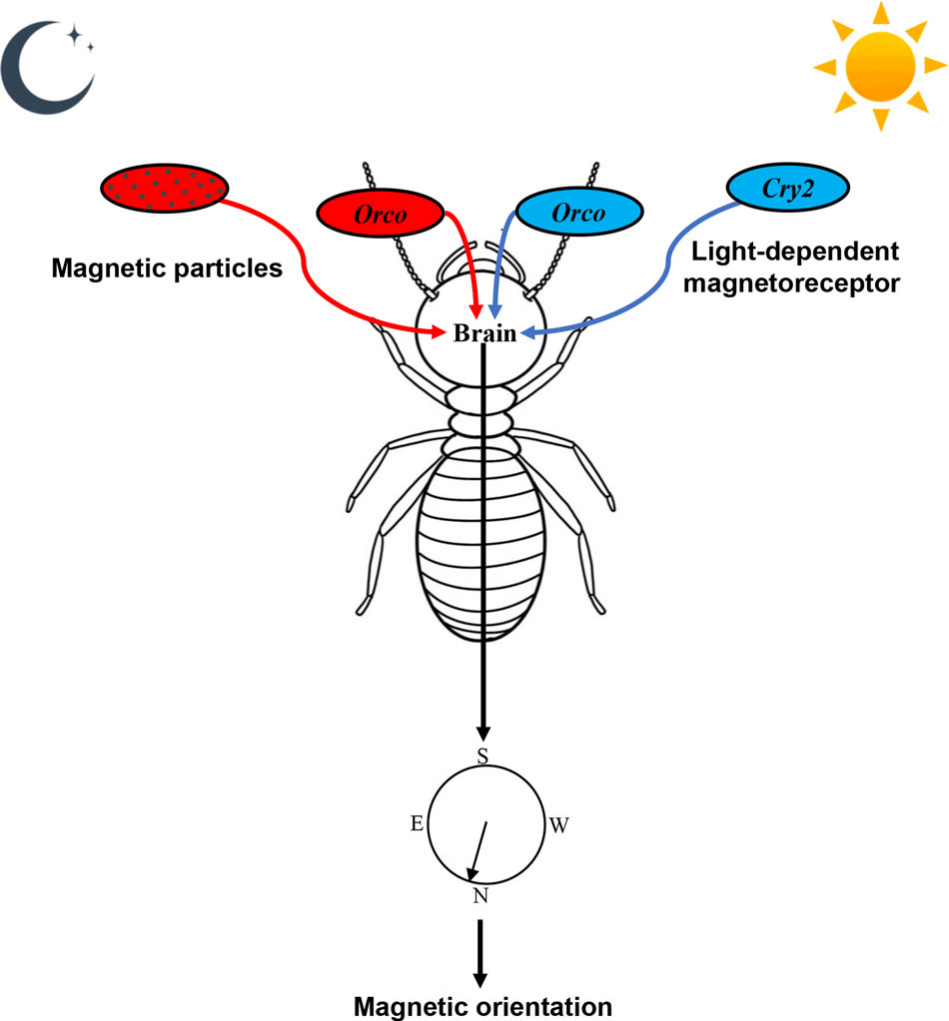
The suggested model for termite orientation involved in magnetoreception mechanism. The termites exhibited directional preference under the GMF in white light and complete darkness. At the molecular level, GMF information is detected by termite Cry2 only in white light, however, GMF information is detected by magnetic particles in termites in complete darkness. In addition, Orco participated in termite magnetic orientation under both light and complete darkness. The joint action of Cry2, magnetic particles, and olfactory co-receptor in regulating magnetic orientation in termites.

In conclusion, termites can use the GMF for orientation in light and darkness. Magnetic orientation for termites shifts from *Cry*2-based mode in light to magnetic particle-based mode in darkness. Strikingly, the *Orco* participates in termite magnetic orientation under light and darkness. Here, we offer a novel magnetoreception model depending on the joint action of radical pair, magnetic particle, and olfactory co-receptor (Fig. 7), which helps further understanding of magnetic orientation.

## Methods

### Experimental termites

The colonies of *R. chinensis* and *O. formosanus* were collected from Shizi Hill, Wuhan City, Hubei Province, China (30°28′N, 114°21′E; GMF strength (49.86 ± 0.15) µT; the horizontal component of the GMF (33.93 ± 0.13) µT (https://www.ngdc.noaa.gov/geomag-web/)). Nine colonies of *R. chinensis* and 34 colonies of *O. formosanus* were used in this study (Supplementary Tables 4 and 5). These colonies were transported to the Insect Behavior Laboratory (IBL) of Huazhong Agricultural University (HZAU), Wuhan, China, and housed in plastic boxes (30 × 15 × 15 cm) containing damp vermiculite. The experimental termites were reared at 25 ± 1 °C and 70 ± 5 % relative humidity (RH) in full darkness. The termites were maintained on and fed with pieces of moist round filter paper (moisture content (MC) 150 %; 15 cm in diameter (id)) for 3 days before the experiment to eliminate the memory of the GMF orientations associated with any food reward. Only worker termites were used for the experiments.

### Helmholtz two-coil system

Custom-built Helmholtz two-coil systems (Fig. 1a, Forever Elegance, CN) were modified as described by Kirschvink^64^ and Fleischmann et al.^10^ to generate the expected AMF. Each coil was a square in shape (side length of 100 cm), and the two coils were placed 51 cm apart, The Helmholtz two-coil system offers a homogeneous magnetic field along an axis through the coil centers (Fig. 1b). The systematic error in the magnetic field strength on the experimental platform (30 cm × 30 cm in 51 cm height) was less than 5%. The coils were connected to a DC power supply (KSP6010) supply to produce a homogeneous magnetic field. The magnetic field could be altered by changing the strength of the current and the direction of the Helmholtz coil system. According to the superposition principle, the combined magnetic field was then obtained by vector addition of the AMF and the GMF. The horizontal component of the magnetic field parameters on the experimental platform was measured using a fluxgate magnetometer (FE-103, Huaming, CN, sensitivity: ±1 nT) before each experiment.

### Magnetic response assays

To assess the horizontal orientation of the worker termites, the moist filter paper (30 cm id) was placed in an open arena in a PVC cylinder dish (30 cm id and 1 cm in height). In each experiment, moist filter papers (2 cm id) containing 7.5% glucose-water were placed at four positions (0°, 90°, 180°, and 270° clockwise relative to the GMF direction) at the edge of the cylinder dish. The dish was placed on a platform (Fig. 1a) in the center of the coil system where the magnetic field was homogeneous. The termites were starved for 12 h before the magnetic response assays^65^. During each experiment, one termite was introduced into the open arena, and its walking behavior was recorded with an infrared video camera (acA1920-40gc, Basler, Germany) at 25 frames/s. Individual termites deprived of social context exhibited “hyperactive” rapid walking behavior when introduced to the test arena. Therefore, we recorded the termite’s walking behavior for 4 min but only analyzed the last 2 min (Supplementary Fig. 3). Heading and turn angle during walking were calculated using the EthoVision video-tracking system (EthoVision XT, Noldus, The Netherlands)^66^. The test conditions of the test environment were as follows: 25 ± 1 °C, 70 ± 5 % RH in either white light (full spectrum light, Fig. 1c) or in complete darkness (red light illumination, Fig. 1d)^67^.

### The detection of magnetic particles

Whole bodies of termite workers were tested for the presence of magnetic particles. Workers of both termite species were extensively washed with 75% (v/v) ethanol and conserved in 75% (v/v) ethanol up to the moment when tested. Then, the samples were dried at 35 °C for 10 h followed by 39 °C for 21 h^68^, maintained inside 1.5 ml plastic centrifuge tube, and ground into a fine powder with tissue grinding pestles. Subsequently, the grinded samples were transferred to a cellulose capsule, and their magnetization hysteresis loops were measured at room temperature using a vibrating sample magnetometer (VSM) (Lakeshore, USA, Model 8600). The loops consisted of a ramp between 20 kOe to −20 kOe and back to 20 kOe for *R. chinensis* and *O. formosanus*. The detection of magnetic particles had three replicates for *R. chinensis* and *O. formosanus*, respectively. Background measurements with empty cellulose capsules were conducted under the same condition, and then their values were subtracted.

### Trail-following bioassays

To assess whether the termites were sensitive to trail pheromones, trail-following bioassays were conducted. (*Z*, *Z*, *E*)-dodeca-3,6,8-trien-1-ol (DTE) and (*Z*, *Z*)-dodeca-3,6-dienol (DDE) are the major compounds present in the trail-following pheromones of *R. chinensis* (Supplementary Fig. 4) and *O. formosanus*^69^, respectively. The Y-shaped trail-following bioassays were performed in an open arena on moist filter paper (MC 150%) with 120° between each branch, on the stem (3 cm) and/or one of the Y branches (5 cm), and trails were drawn with a 5 μL syringe containing DTE, DDE (concentrations see Supplementary Tables 1 and 2) or hexane as a control^69,70^. A termite was introduced into a release chamber (plastic vial of 3 cm id with a 2 mm-wide opening) from which it was allowed to walk on the filter paper to follow the scented trail. Trails being 8 cm with turns at an intersection followed by termites were considered to be highly active (selection test score = 2). Trails longer than 3 cm with turns at an intersection were considered to be less active (selection test score = 1), and those shorter than 3 cm were considered to be inactive (selection test score = 0)^41^. The filter paper and the tested termites were used only once. Every bioassay involved at least 15 termites.

### Orientation test of the interaction between the magnetic field and trail pheromone

To simulate the highly branched trail pheromone conditions within a termite nest, filter paper impregnated with 8 equal doses, with 10-cm-long pheromone trails (DTE (1 pg/cm) for *R. chinensis* and DDE (0.1 pg/cm) for *O. formosanus*, was used as the open arena in the dish-like cylinder arena as described above. The trails were spread out radially from the center of the circular device^20^. The device was placed in the center of the Helmholtz coil system for video recording as described above.

### Quantitative real-time PCR (qRT-PCR)

qRT-PCR was used to assess the effect of knockdown treatment on targeted gene expression, and the expression levels of the targeted genes were observed in different organs of the termites. Total RNA was extracted from the heads of 10 worker termites using RNAiso Plus (Takara, Japan). One microgram of total RNA was used for cDNA synthesis following the instructions of the PrimeScript RT reagent Kit with gDNA Eraser (Takara, Japan). Then, qRT-PCR was performed with the corresponding primers (Supplementary Table 6) and SYBR Green Master Mix (YEASEN, China) using a QuantStudio 6&7 Flex Real Time PCR System (Applied Biosystems, Life Technologies, Milan, Italy). All data were normalized to the relative mRNA levels of reference genes (*β-actin*, *Hsp 70*, or *NADH*). The primer sequences used for qRT-PCR are listed in Supplementary Table 6. For each qRT-PCR assay, there were at least eight independent biological replicates. The 2^−ΔΔCT^ method was used to analyze the qRT-PCR data^71^.

### Synthesis of dsRNA and microinjection

The dsRNAs were synthesized from plasmids containing the appropriate gene fragments (see Supplementary Table 7 for primer sequences) with the T7 transcription kit (Fermentas, Lithuania). The dsRNA quality was verified by electrophoresis in a 1% native agarose gel, and the concentration was measured with a NanoDrop 2000 spectrophotometer (Thermo Scientific, Wilmington, DE, USA). A sterilized microinjector (SYS-PV820, World Precision Instruments, USA) was used to inject 2 µg of a 15-20 µg/µl dsRNA solution into the side of the thorax of ice-anesthetized worker termite^72^. After dsRNA injection, the termites were placed on moist filter paper (MC 150%) within a 9-cm Petri dishes. The heads of the workers were collected for the mRNA analysis of *Cry*2, *MagR* or *Orco* after injection. Controls were injected with an equivalent amount of double-stranded green fluorescent protein (ds*GFP*)^73^.

### Orientation test under an orthogonal design

We used an orthogonal design L9 (3^4^) to analyze the effect of *Orco* (noninjected, ds*GFP*-injected and ds*Orco*-injected), *Cry*2 (noninjected, ds*GFP*-injected and ds*Cry*2-injected), trail pheromone (DTE (0, 0.01 and 1 pg/cm) for *R. chinensis* and DDE (0, 0.001 and 0.1 pg/cm) for *O. formosanus*) and magnetic field strengths (elimination, GMF, 0.1 mT) on the orientation in the two termite species (Supplementary Table 8). The orientations of treated worker termites were recorded and analyzed using the methods described above.

### Statistics and reproducibility

For angle values, circular statistics were performed using Oriana 4.0 (KCS, Pentraeth, UK) (https://www.kovcomp.co.uk/oriana/ oribroc.html), and the mean direction was tested using a Rayleigh test. Metric data were analyzed using SPSS Statistics 19.0 (IBM, US). The normal distribution of the data was tested via the Shapiro-Wilk method in each analysis. The effects of the sampled termite organs on the expression levels of the *Cry*2 and *MagR* genes were analyzed using one-way analysis of variance (ANOVA), and differences were compared using Tukey’s HSD tests. Magnetic parameters of two termite species were analyzed using independent-samples *t*-tests. Alternatively, the Wilcoxon test and Mann–Whitney test were used to analyze the abnormally distributed data. The effect of ds*MagR*, ds*Cry*2 or ds*Orco* injections on the expression of targeted genes was analyzed using the Wilcoxon test. The effects of ds*MagR*, ds*Cry*2 or ds*Orco* injection on the walking turn angle of *O. formosanus* an*d R. chinensis* were analyzed using the Mann–Whitney test. Additionally, the effects of ds*Orco*-injection on the pheromone test score were evaluated using the Mann–Whitney test. In all experiments, the significance level was set as *p* = 0.05. In addition, the number of replicates is indicated in individual figure and the methods.

### Reporting summary

Further information on research design is available in the Nature Research Reporting Summary linked to this article.

## Supplementary information

Supplementary Information

Description of Additional Supplementary Files

Supplementary Movie 1

Supplementary Movie 2

Supplementary Movie 3

Supplementary Movie 4

Supplementary Data 1

Reporting Summary

## Data Availability

All data generated and analyzed for this study are included in the article, Supplementary Data 1 and Supplementary Information files. Any remaining information can be obtained from the corresponding author upon reasonable request.
