# Supplementary material for: In addition to cryptochrome 2, magnetic particles with olfactory co-receptor are important for magnetic orientation in termites
Source: Commun Biol. 2021 Sep 23;4:1121. doi: 10.1038/s42003-021-02661-6 (PMC8460727; doi:10.1038/s42003-021-02661-6)
Supplement: Supplementary file 1 — Supplementary Information [file 42003_2021_2661_MOESM1_ESM.pdf]

Supplementary Information for

In addition to cryptochrome 2, magnetic particles with olfactory co-receptor are important for magnetic orientation in termites

Yongyong Gao[1], Ping Wen[2], Ring T Cardé[3], Huan Xu[1], Qiuying Huang[1*]

Supplementary Information

Supplementary Date 1: Data underlying figures and supporting information figures.

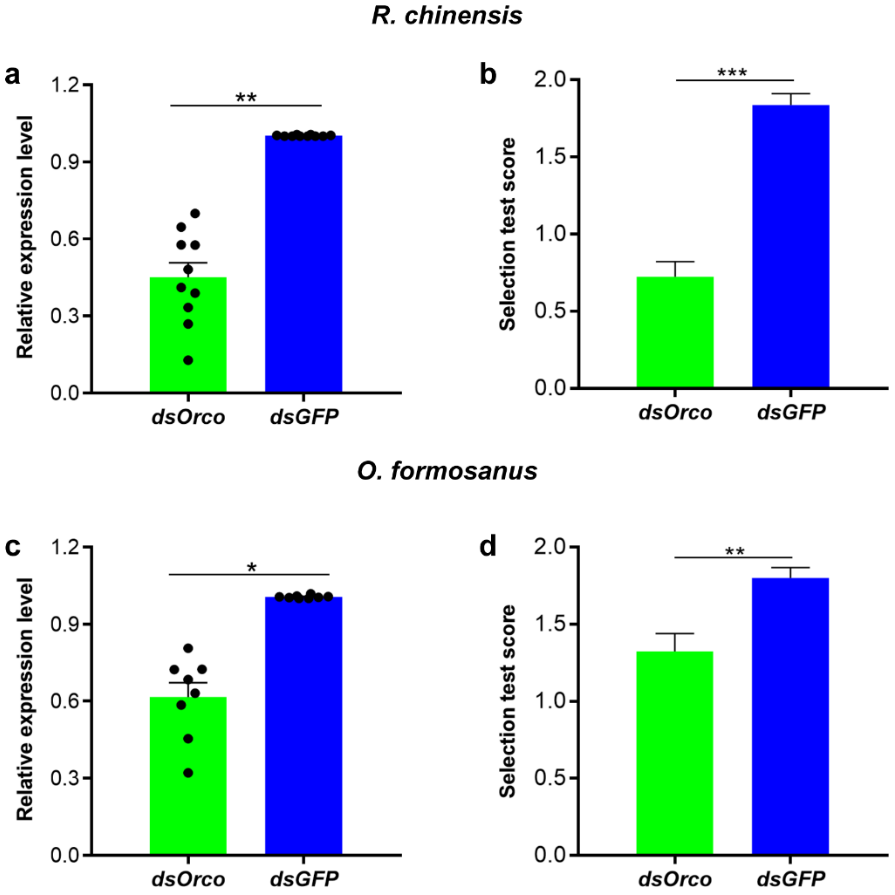

Supplementary Fig. 1. Pheromone selection following the knockdown of *Orco* in *R. chinensis* and *O. formosanus*. **a** The *Orco* mRNA levels in *R. chinensis* head 5 days after injection of ds*Orco* and ds*GFP* (n = 10). **b** Pheromone selection test score in *R. chinensis* 5 days after the injection of ds*Orco* (n = 47) or ds*GFP* (n= 37). **c** *Orco* mRNA levels in the *O. formosanus* head 3 days after the injection of ds*Orco* and ds*GFP* (n=8). Bars represent the mean ± SEM. Asterisks indicate significant differences determined by the Wilcoxon test (*, $p < 0.05$; **, $p < 0.01$) (**a**, **c**). **d** Pheromone selection test score in *O. formosanus* 3 days after the injection of ds*Orco* (n = 37) or ds*GFP* (n = 35). Bars show the mean scores ± SEM. Asterisks indicate significant differences determined by the Mann-Whitney test (**, $p < 0.01$; ***, $p < 0.001$) (**b**, **d**).

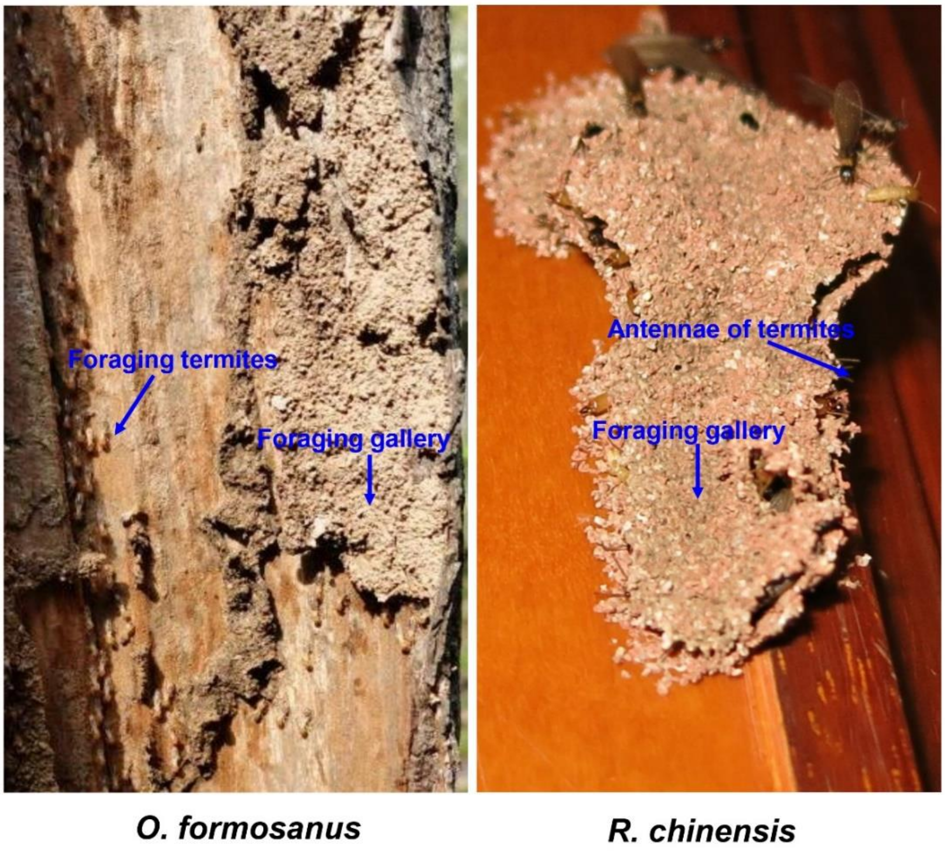

Supplementary Fig. 2. The foraging termites of *R. chinensis* and *O. formosanus* can receive white light beneath the soil surface under natural conditions because the light can go through the cracks and pores of foraging galleries.

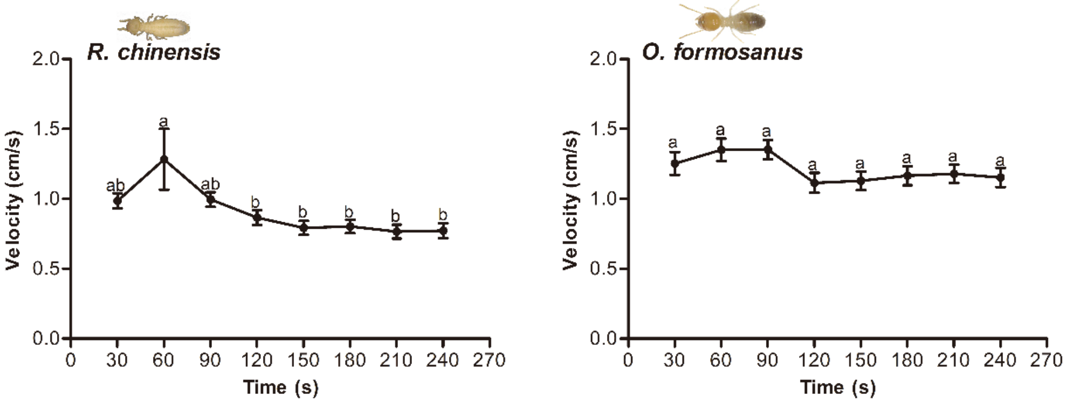

Supplementary Fig. 3. Walking velocity of *R. chinensis* and *O. formosanus* in the test arena for 4 minutes. The data in the figures are the mean ± SEM. The normal distribution of data was tested by the Shapiro-Wilk method. The walking velocity of *R. chinensis* (n = 27) and *O. formosanus* (n = 31) was analyzed using one-way analysis of variance (ANOVA), and different letters express significant differences according to Tukey's HSD test (*ANOVA*, *R. chinensis*: $F_{(7,\ 208)} = 3.81$, $p < 0.001$; *O. formosanus*: F(7, 240) = 1.79, p = 0.09).

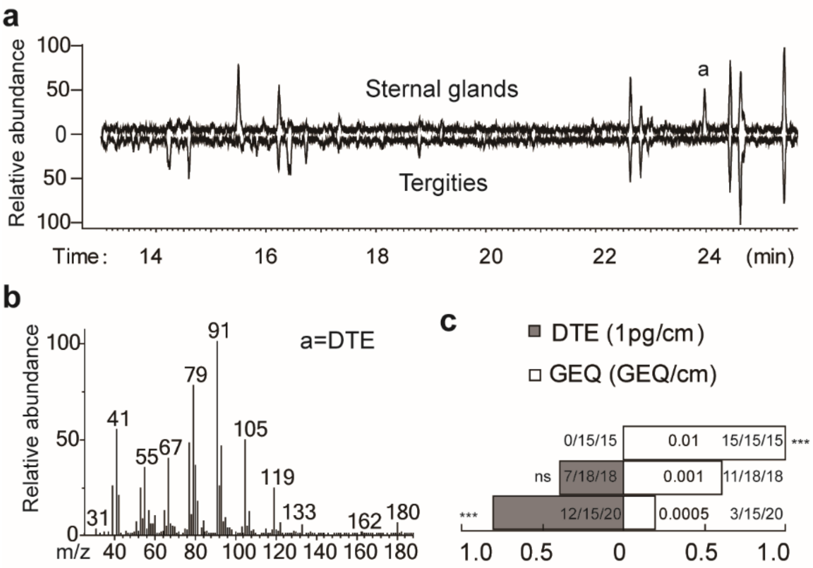

Supplementary Fig. 4. Identification of the trail pheromone of *R. chinensis*. a (*Z*, *Z*, *E*)-dodeca-3,6,8-trienol (DTE) was specific on the sternal gland surface. A signal SPME extract form 200 workers was analyzed on an HP6890-5975C GC-MS with an HP-5ms column. **b** The mass spectrum was identical to that of the synthetic trail pheromone. **c** The preference of *R. chinensis* workers for the sternal gland extract and 1 pg/cm synthetic dodecatrienol indicated that the gland extract may contain 0.5 ng to 1 ng dodecatrienol. The asterisk indicates that the selection was significant (triple asterisks indicated $p < 0.001$, while a double asterisk indicated $0.001 < p < 0.01$). “ns” indicates that the selection was not significant.

Supplementary Table 1. Pheromone selection test of DTE-treated workers of *R. chinensis*

DTE (pg/cm)	Active sample	Control	N	*Sig*
10.00	16	0	16	***
1.00	16	0	16	***
0.10	13	0	15	**
0.01	3	0	16	ns

Selections were analyzed by the Mann-Whitney test with continuity correction (***, $p < 0.001$; **, $p < 0.01$; ns, no significant).

Supplementary Table 2. Pheromone selection test of DDE-treated workers of *O. formosanus*

DDE (pg/cm)	Active sample	Control	N	*Sig*
1.00	7	0	16	ns
0.10	15	0	15	***
0.01	11	0	15	**
0.001	2	0	15	ns

Selections were analyzed by the Mann-Whitney test with continuity correction (***, $p < 0.001$; **, $p < 0.01$; ns, no significant).

Supplementary Table 3. Magnetic parameters of *R. chinensis* and *O. formosanus*

**Species**	***Js* (memu)**	***Hc* (Oe)**	***Jrs* (memu)**	***Jrs/Js***
R. chinensis	1.21 ± 0.22	104.25 ± 0.03	0.13 ± 0.03	0.11 ± 0.002
O. formosanus	3.74 ± 0.65	102.05 ± 2.12	0.38 ± 0.09	0.10 ± 0.006

Supplementary Table 4. The distribution of the 9 colonies of *R. chinensis* for each experiment*

Experiments	Replicates	Colonies
Magnetic orientation	19-24	Number 1, 2, 3, 4
Trail-following bioassays	15-16	Number 1, 5, 7
Magnetic orientation for pheromone	40-59	Number 1, 2, 3, 4
Expression of *MagR* and *Cry2*	9	Number 5, 6
RNAi efficiency for *Cry2*	9	Number 7, 8, 9
RNAi efficiency for *MagR*	9	Number 7, 8, 9
Orientation behavior for *Cry2*	43	Number 7, 8, 9
Orientation behavior for *MagR*	46	Number 7, 8, 9
RNAi efficiency for *Orco*	10	Number 2, 3, 4
Orientation behavior for *Orco*	37	Number 1, 2, 3
RNAi efficiency for pheromone selection	47	Number 2, 3, 4
Orthogonal design	54-67	Number 1, 2, 3, 4
Detection of magnetic particles	3	Number 1, 2, 3

* All 9 colonies of *R. chinensis* were collected from Shizi hill in Wuhan City, Hubei Province, China.

Supplementary Table 5. The distribution of the 34 colonies of *O. formosanus* for each experiment *

Experiments	Replicates	Colonies
Magnetic orientation	15-23	Number 1, 2, 3, 4
Trail-following bioassays	15-16	Number 5, 6, 7
Magnetic orientation for pheromone	40-41	Number 8, 9, 10
Expression of *MagR* and *Cry2*	8	Number 11,12, 13
RNAi efficiency for *Cry2*	9	Number 14, 15, 16
RNAi efficiency for *MagR*	9	Number 14 ,15, 16
Orientation behavior for *Cry2*	38	Number 17, 18, 19, 20
Orientation behavior for *MagR*	35	Number 17, 18, 19, 20
RNAi efficiency for *Orco*	8	Number 20, 21, 22, 23
RNAi efficiency for pheromone selection	37	Number 20, 22, 23
Orientation behavior for *Orco*	44	Number 24, 25, 26, 27
Orthogonal design	41-57	Number 28, 29, 30, 31
Detection of magnetic particles	3	Number 32, 33, 34

* All 34 colonies of *O. formosanus* were collected from Shizi hill in Wuhan City, Hubei Province, China.

Supplementary Table 6. Primers used for qRT-PCR analyses

Species	Gene Name	Orientation	(5′→3′) Primer Sequence
R. chinensis	*β-actin*	Forward	AGCGGTCACTCATTCCCTTG
		Reverse	ATTCCTGACGTACTGTCGCC
	Hsp 70	Forward	GCTAAGCGTCTTATTGGGCG
		Reverse	CTTTGGTTTTCCACCGTCGC
	Cry2	Forward	AGTTCTTTGCACATGGCCG
		Reverse.	CGTGAAGGACTGAACTCTGCT
	MagR	Forward	AACGTGTGGCTGTGGTGAAA
		Reverse	CTGTTGACACATTCCTTCTGCC
	Orco	Forward	TGGTATGACGGTTCGGAGGAA
		Reverse	ACCAGCACCATGAAGTAGGTC
O. formosanus	*β-actin*	Forward	CTGGAGAAGTCATACGAGTTG
		Reverse	AGAAGGAAGGCTGGAACA
	NADH	Forward	TTGGTGAGATTGGTCTGCTG
		Reverse	ACAATGTGTAAGCCGCACTA
	Cry2	Forward	TCTTACCCGTGGCGATCTCT
		Reverse	AAGCGCACTGGACAATAGCA
	MagR	Forward	ATGGCGAGCCGTATTGTATC
		Reverse	CTGTTGACACATTCCTTCTGCC
	Orco	Forward	TGGTCATGTACGTGGGCATC
		Reverse	CCGCCAGACATAGCATTCCA

Supplementary Table 7. Primers used for cloning the dsRNA template

Species	Gene Name	Orientation	(5′→3′) Primer Sequence
R. chinensis	*Cry2*	Forward	GAGGTGTCATTTTAGGGCGA
		Reverse	TTCAAGGAATGGGGCACTAC
	MagR	Forward	AGAATCGCAAGTGCCAGAGT
		Reverse	CACAGCCACACGTTCCTTTA
	Orco	Forward	ACCGTGAATGTGTATGCCTG
		Reverse	AATAGCACCCAACACCGAAG
	GFP	Forward	CTTGAAGTTGACCTTGATGCC
		Reverse	TGGTCCCAATTCTCGTGGAAC
O. formosanus	*Cry2*	Forward	TCGCCCTAAAATGACACCTC
		Reverse	CACCACGGGTAAGAAAGCAT
	MagR	Forward	ATGGCGAGCCGTATTGTATC
		Reverse	TCTGACGACAGTTGCGTTTC
	Orco	Forward	TAACTCCGCAGACCTGTTCC
		Reverse	GAATTTGGCTCCAGAGATGC
	GFP	Forward	CTTGAAGTTGACCTTGATGCC
		Reverse	TGGTCCCAATTCTCGTGGAAC

Supplementary Table 8. The comprehensive effect of *Orco*, *Cry2*, the trail pheromone and magnetic field strength on the orientation of *R. chinensis* and *O. formosanus*

**Species**	**Treatment**	***Orco***	***Cry2***	**Trail pheromone (pg/cm)**	**Magnetic field strength**
R. chinensis	1	Noninjected	Noninjected	0	Elimination
	2	Noninjected	ds*GFP*	0.01	GMF
	3	Noninjected	ds*Cry2*	1	0.1 mT
	4	ds*GFP*	Noninjected	0.01	0.1 mT
	5	ds*GFP*	ds*GFP*	1	Elimination
	6	ds*GFP*	ds*Cry2*	0	GMF
	7	ds*Orco*	Noninjected	1	GMF
	8	ds*Orco*	ds*GFP*	0	0.1 mT
	9	ds*Orco*	ds*Cry2*	0.01	Elimination
O. formosanus	1	Noninjected	Noninjected	0	Elimination
	2	Noninjected	ds*GFP*	0.001	GMF
	3	Noninjected	ds*Cry2*	0.1	0.1 mT
	4	ds*GFP*	Noninjected	0.001	0.1 mT
	5	ds*GFP*	ds*GFP*	0.1	Elimination
	6	ds*GFP*	ds*Cry2*	0	GMF
	7	ds*Orco*	Noninjected	0.1	GMF
	8	ds*Orco*	ds*GFP*	0	0.1 mT
	9	ds*Orco*	ds*Cry2*	0.001	Elimination
