# Supplementary material for: In addition to cryptochrome 2, magnetic particles with olfactory co-receptor are important for magnetic orientation in termites
Source: Commun Biol. 2021 Sep 23;4:1121. doi: 10.1038/s42003-021-02661-6 (PMC8460727; doi:10.1038/s42003-021-02661-6)
Supplement: Supplementary file 2 — Description of Additional Supplementary Files [file 42003_2021_2661_MOESM2_ESM.pdf]

Description of Additional Supplementary Files

File name: Supplementary Movie 1

Description: Walking behavior of dsCry2- and dsGFP-injected R. chinensis under the GMF in white light.

File name: Supplementary Movie 2

Description: Walking behavior of dsCry2- and dsGFP-injected O. formosanus under the GMF in white light.

File name: Supplementary Movie 3

Description: Walking behavior of dsOrco- and dsGFP-injected R. chinensis under the GMF in white light.

File name: Supplementary Movie 4

Description: Walking behavior of dsOrco- and dsGFP-injected O. formosanus under the GMF in white light.

File name: Supplementary Data 1

Description: Data underlying figures and supporting information figures.
